# Community awareness and neuroepidemiology of onchocerciasis-associated epilepsy in two rural communities in Cameroon

**DOI:** 10.11604/pamj.2025.50.42.45546

**Published:** 2025-02-04

**Authors:** Mundih Noelar Njohjam, Mark Olivier Ngoule, Emanuelle Mylene Tonga, Annick-Sandra Jouonang Teugang

**Affiliations:** 1 Department of Neurology, Cheikh Anta Diop University, Dakar, Senegal

**Keywords:** Onchocerciasis-associated epilepsy, prevalence, incidence, community awareness

## Abstract

**Introduction:**

onchocerciasis is the leading cause of epilepsy in onchocerciasis-endemic communities. Our study aimed to determine the incidence, prevalence and community awareness of Onchocerciasis-associated epilepsy (OAE) in two onchocerciasis-endemic rural communities in Cameroon.

**Methods:**

we conducted a community-based cross-sectional study in two rural villages (Yangafock I, and Yangafock II) in the Mbam et Kim division of the Center Region of Cameroon. Door-to-door household visits were conducted to screen for individuals with epilepsy. OAE was defined using previously established criteria. Using an established questionnaire, we assessed community awareness of OAE.

**Results:**

seven hundred and ninety-nine (799) peoples from 102 households (380 in Yangafock I and 419 in Yangafock II) were screened. Fifty-three (53) peoples, (33 from Yangafock I and 20 from Yangafock II) met the OAE clinical criteria. Two hundred and forty-nine (249) persons completed the community awareness questionnaire. For Yangafock I, the overall prevalence of epilepsy was 78.75 per 1000, while for Yangafock II, it was 47.7 per 1000. The five-year incidence of epilepsy was 23.8 per 1000 and 11.9 per 1000 for 44 Yangafock I and Yangafock II, respectively. The most affected age group was 20-29 years. Community members had a critically low level of OAE awareness and risk perception.

**Conclusion:**

the high prevalence and a critically low level of public awareness underscore the need to raise community awareness of OAE to increase community engagement in the fight against onchocerciasis and its complications.

## Introduction

Onchocerciasis, a parasitic infection commonly known as river blindness, is a neglected tropical disease caused by the filarial worm *Onchocerca volvulus*, which is transmitted through the bites of infected blackflies of the genus Simulium [1]. It is an endemic disease, with the vast majority of cases located in sub-Saharan Africa (SSA) [1,2]. Until recent years, blindness was the most feared complication of onchocerciasis [1]. There is a growing body of evidence suggesting that onchocerciasis may induce seizures and epilepsy in people with onchocerciasis living in regions where the disease is endemic [3-11]. This form of epilepsy is known as onchocerciasis-associated epilepsy (OAE) [3]. Although the underlying mechanisms of OAE are not fully understood, several hypotheses currently exist. One hypothesis suggests that the release of retinoids from dying microfilariae (the larval stage of the parasite) and their gradual accumulation to toxic concentrations in affected tissues may play a role [1]. The parasite may also induce seizures through the migration of microfilariae or the inflammatory response to the dying worms [1]. Two cohort studies done in Cameroon found that having a higher microfilarial load as a child is linked to a higher risk of getting epilepsy later in life [12,13]. Clinical criteria for diagnosing OAE have been developed and validated [3]. Table 1 summarizes the clinical criteria for OAE.

**Table 1:** summary of proposed case definitions for onchocerciasis-associated epilepsy

OAE, minimal required criteria (2)	Persons who satisfy the following six criteria: (1) a history of two or more unprovoked epileptic seizures occurring at least 24 h apart; (2) living in an onchocerciasis-endemic region for at least three years; (3) living in a village with a high prevalence of epilepsy and persons with epilepsy often clustered within certain households, that is, families having more than one child with epilepsy; (4) no other obvious cause of epilepsy (e.g. perinatal asphyxia, history of severe malaria, measles, encephalitis or meningitis, or head injury with loss of consciousness in the five-years before the onset of epileptic seizures); (5) onset of seizures in childhood or adolescence (3 to 18 years); (6) normal neurological development before the onset of epilepsy.
Additional criteria suggesting OAE (3)	(1) a history of head nodding seizures, cognitive impairment or Nakalanga features+ (2) persons with these features in the same village (3) seropositivity for Ov16 onchocerciasis antibodies (4) for a person who has never taken ivermectin: a. skin test positivity for microfilaria (microscopy or PCR***) b. clinical manifestations of onchocerciasis, including leopard skin, onchodermatitis, ocular onchocerciasis and/or nodules.
New additional criterion suggesting OAE	Knowledge that the area is or was onchocerciasis meso- or hyperendemic based on > 20% nodule prevalence in REMO** surveys or > 35% prevalence of microfilaria in the skin

The burden of onchocerciasis and OAE in SSA is huge with significant economic and social impact, as it often affects young individuals, leading to disability, reduced quality of life, and significant healthcare costs [11]. Current evidence suggests that reducing community microfilaria load through ivermectin distribution can delay or prevent the development of seizures in onchocerciasis-infected persons living in onchocerciasis-endemic communities [7,14,15]. Several studies conducted in different countries in SSA have reported a significant reduction in the incidence of OAE after reinforcement of measures for onchocerciasis elimination, such as community distribution of ivermectin [11,14,15].

Ivermectin is an extremely effective antiparasitic drug that has been the main preventive strategy in the fight against onchocerciasis [1]. Mass drug administration with ivermectin has contributed to the tremendous progress made towards global elimination of onchocerciasis [1]. However, there are still several challenges that threaten efforts towards elimination, including poor community participation in elimination efforts. A lack of community participation, such as in taking ivermectin has been consistently reported as a barrier to the elimination of onchocerciasis and has been attributed to low community awareness of onchocerciasis [1,16,17]. Conversely, a high level of community awareness of onchocerciasis has been associated with increased uptake of ivermectin [1,16,17]. The scientific community is increasingly informed by new evidence on the existence of a causal relationship between onchocerciasis and epilepsy. However, the people who are affected by OAE or are at increased risk may not be aware.

Data on community awareness of OAE is sparse in SSA. The main objective of this study was to assess the level of community knowledge or awareness of OAE in two rural onchocerciasis foci in Cameroon. Additionally, we aim to describe the neuroepidemiology of OAE in these communities. Assessing and addressing OAE knowledge gaps could improve community ivermectin uptake and participation in the fight against onchocerciasis. As has been done in many communities where onchocerciasis is common, data on the incidence and prevalence of OAE could also push policymakers or local stakeholders to strengthen current strategies and start developing new ones to ensure elimination of the disease.

## Methods

### Study site and population

**Figure 1:**
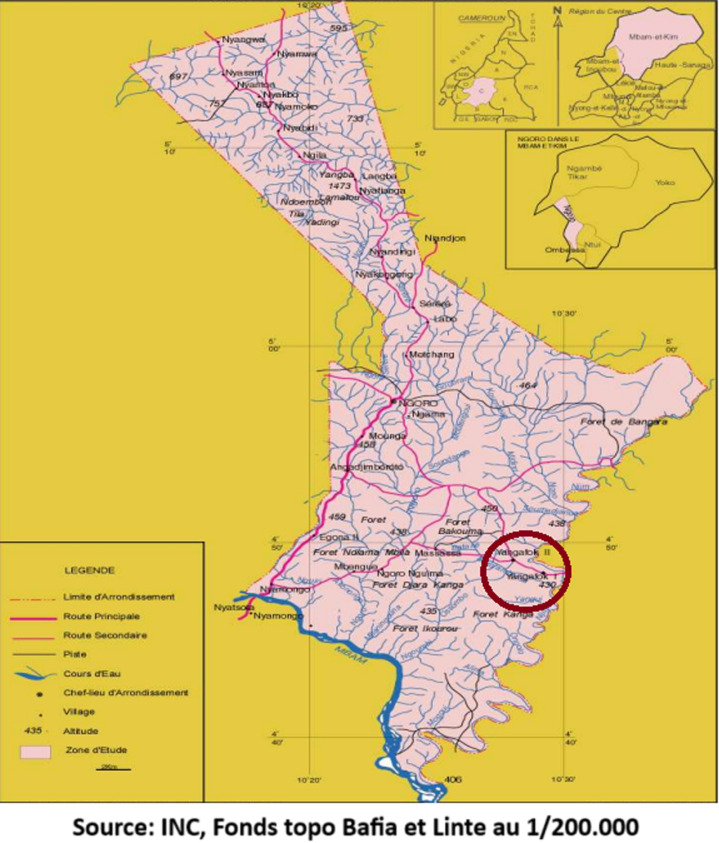
map showing study sites (encircled)

The study was carried out in two rural villages situated in the Ngoro subdivision of the Mbam et Kim division in the Centre Region of Cameroon; Yangafock I and II (Figure 1). The Mbam et Kim division is known to be a hyperendemic focus for onchocerciasis [4]. This is probably because of the presence of fast-flowing rivers such as the Mbam river (which is the largest tributary of the Sanaga river), rivers Ngoro, Mbi and Kenkeng. The many rapids of the Mbam River as well as the Ngoro River are excellent breeding grounds for the black flies, as they provide the oxygenated water that is indispensable for the survival of the larvae of the black fly. Onchocerciasis prevalence is reported to be high in villages along the Mbam River. Yangafock I and II are two small rural villages that are located close to the Mbam River (Figure 1). Yangafock I has a total population size of about 380 while Yangafock II has a total population estimated to be about 1200. The main income-generating activity of the people is agriculture. Other income-generating activities include fishing, animal rearing, and sand extraction. Community-directed treatment with ivermectin is done annually but not regularly due to the non-availability of the drug, as reported by some of the inhabitants.

### Sample size calculation

The following formula was used to determine the minimum sample size for the neuroepidemiology component of the study:


$$n=Z^{2}\times p\left(1-p\right)/d^{2}$$

Where: n = the required sample size, Z is the Z-score representing the number of standard deviations from the mean, which is 1.96 for a 95% confidence level, p= the expected prevalence of the population, d= desired degree of accuracy.

A previous study reported a prevalence of 7.8% of OAE in a rural community in Cameroon [4]. We used 5% for the degree of accuracy (d).

With these assumptions, the minimum required sample size n = [[(1.96)^2^ × 0.078(1-0-078)]/(0.05)^2^] for the neuroepidemiology component was 111 participants. We used the same sample size formula to calculate the minimum sample size for the community awareness component of the study but with the following assumptions:

We reviewed the literature for studies on community awareness of OAE, but we didn´t find any similar studies. We therefore estimated a 50% level of community awareness, 5% for the degree of accuracy (d) and 95% confidence interval. Based on these assumptions, the minimum required sample size n= [[(1.96)^2^ × 0.5(1-0.5)]/ (0.05)^2^] for the community awareness component was 384.

### Study design and period

This was a community-based cross-sectional study conducted from May 1^st^ to May 31^st^, 2021. We obtained authorizations to conduct the study from each local chief before carrying out study activities in each village, detailing the objectives, procedures, and implications of the research. Each local chief assigned community relay agents to sensitize the entire village before conducting door-to-door visits. These relay agents also assisted the research team in gaining access to the study participants' homes. We conducted door-to-door visits in each village to identify cases of OAE and recruit participants for the knowledge assessment. During house-to-house visits, the study's objectives and procedures were explained to each household head or his representative, as well as the final respondent. Written informed consent was obtained from the head of each household, as well as the suspected case of epilepsy.

### Identification of onchocerciasis-associated epilepsy cases

The OAE cases were identified in multiple stages.

The first stage was the general screening for epilepsy. The general screening was done using a five-item questionnaire validated for epilepsy screening in tropical regions [18]. The five screening questions contained in the questionnaire were: have you ever lost consciousness and experienced either loss of bladder control or foaming in the mouth? Have you ever experienced absence or sudden loss of contact with the surroundings for a short duration of time? Have you ever experienced sudden, uncontrollable twitching or shaking of the arms, legs, or head for a period of a few minutes? Do you sometimes experience sudden and brief bodily sensations, see or hear things that are not there, or smell strange odours, etc.? Have you ever been diagnosed with epilepsy?

The second stage was interrogation and neurological/clinical assessment of suspected cases of epilepsy identified during the general screening by epilepsy-trained physicians. For history taking and general clinical assessment, a standard patient-clerking questionnaire was used to collect data on sociodemographic data, past medical history, clinical data, and physical examination.

In the third stage, the criteria for epilepsy diagnosis proposed by the International League Against Epilepsy (ILAE) were applied to all suspected cases of epilepsy [19]. Based on these criteria proposed by the ILAE, a participant was considered a case of epilepsy if they met at least one of the following: presence of at least two unprovoked (or reflex) seizures occurring greater than 24 hours apart or a single seizure with a probability of recurrence of at least 60%, and having a diagnosis of an epilepsy syndrome.

Finally, the clinical criteria for OAE diagnosis (Table 1) were applied to ensure that only participants with OAE were included in the neuroepidemiology phase of the study.

### Definitions

Incidence Rate= total number of new cases of disease / total population at risk x Population size.

Prevalence (%)= all cases of OAE per village / total number of inhabitants surveyed x 100.

### Community knowledge assessment

During the door-to-door visits, we invited individuals from the communities to complete a knowledge assessment questionnaire to understand the community's general knowledge of onchocerciasis-associated epilepsy. This was done using a random selection approach, in which community members were randomly chosen from each household visited. We used a randomization method that involved flipping a coin to determine which household member would receive an invitation to participate. In addition to the door-to-door recruitment, we also visited community schools and religious institutions to identify and recruit participants. This ensured that we included a representative sample of the community, including individuals of different ages and socioeconomic backgrounds. The randomly selected participants were given detailed information about the study and invited to take part in the survey. Those who agreed to participate were asked to provide written informed consent before enrolling in the study. Additionally, participants who were identified as cases of OAE in the neuroepidemiology phase were also invited to participate.

In the community awareness phase of the study, we assessed the level of awareness of community members who did not have OAE as well as those who were identified as cases of OAE during the neuroepidemiology phase.

### Inclusion criteria for onchocerciasis-associated epilepsy cases

To be included in the study, participants had to meet all of the following criteria: must meet the diagnostic criteria for epilepsy and OAE; the patient does not have any other identifiable cause of epilepsy; has willingly consented to participate; parental consent has been given to those aged less than 18 years; has been living in the study site at the time of onset of epilepsy. We excluded those who had epilepsy from other causes, as well as those with OAE who did not consent or were unable to respond to questions.

### Inclusion criteria for participants for the community awareness assessment

To be included in the study, participants had to meet all of the following criteria: have willingly consented to participate; parental consent has been given to those aged less than 18 years; must have been a permanent resident in the study site since birth.

We excluded those who did not give informed consent or who were not able to respond to questions. We also excluded those who had recently moved to the study sites.

### Ethical considerations

Ethical approval for this study was obtained from the regional ethical review committee for human research in the Centre Region of Cameroon. All participants were informed of the study's activities and implications of participating in the study and informed consent was obtained prior to enrolment. The study was conducted following the principles of the Declaration of Helsinki. No data that could identify participants such as names and phone numbers were collected.

### Bias

The door-to-door approach and the use of community relay agents who knew the addresses of the different cases allowed us to reach every potential case of OAE in the communities. Also, we made sure that only people with OAE were included by carefully screening and evaluating each participant. This included looking at their medical records, talking to their family members, and strictly applying the criteria for OAE diagnosis. Lastly, the use of local translators to translate questions ensured that patients and their caregivers clearly understood questions before responding. Moreover, the random sampling approach that we used to recruit participants without epilepsy ensured that the sample is representative of the community and minimized selection bias.

### Data management and analysis

Data was collected in the field using paper forms and entered into SPSS version 26.0. All data collection sheets were checked for correctness and completeness. We excluded incomplete forms from the data analysis. Descriptive statistics such as frequencies, mean and standard deviation were used to summarize categorical data. Continuous data were expressed as means ± standard deviation (SD). Frequencies and percentages were used to summarize data on prevalence and incidence. The level of statistical significance was set at p <0.05.

## Results

**Figure 2:**
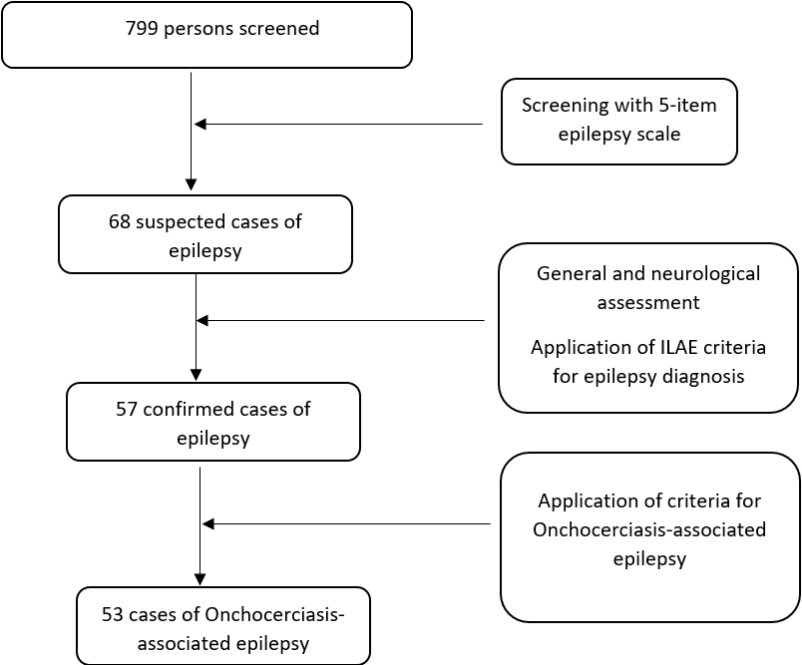
flow chart for the recruitment of participants with onchocerciasis-associated epilepsy

We visited 102 households and screened 799 individuals during the study period, resulting in the identification of 57 confirmed cases of epilepsy (33 from Yangafock I and 20 from Yangafock II). We assessed the fifty-seven cases neurologically and clinically, and confirmed 53 as OAE using the validated clinical criteria for OAE. Four cases had epilepsy from other causes, including complications from meningitis, perinatal injury, and cerebral malaria. Figure 2 summarizes the recruitment process for participants with OAE.

### Sociodemographic profile

There was a predominance of male participants, and most participants were farmers. Primary education was the highest level of educational attainment for most participants. The majority of participants were single. Table 2 summarizes the sociodemographic data of the cases of OAE. One hundred and ninety-six (196) peoples, community members (mean age of 33.85 ± 14) completed the community awareness questionnaire, along with the 53 participants with OAE. Table 3 summarizes the sociodemographic data of the participants without OAE.

**Table 2:** demographic data of participants with onchocerciasis-associated epilepsy recruited from Yangafock I and II villages from May 1^st^ to May 31^st^, 2021 (N=53)

Variable		%	(n)
Sex			
	Male	45.3	24
	Female	54.7	29
	Total	100	53
**Occupation**			
	Farmer	71.7	38
	Trader	7.5	4
	Teacher	1.9	1
	Student	9.4	5
	Unemployed	7.5	4
	Others	1.9	1
	Total	100	53
**Level of education**			
	Primary	73.6	39
	Secondary	43.88	86
	None	7.5	4
**Level of income**			
	< 100 USD per month	66	35
	100-200 USD per month	3.8	2
	201-300 USD per month	9.4	5
	None	20.8	11
	Total	100	53
**Marital status**			
	Single	71.7	38
	Married	28.3	15
	Total	100	53

**Table 3:** demographic data of participants without onchocerciasis-associated epilepsy recruited from Yangafock I and II villages, from May 1^st^ to May 31^st^, 2021 (N=196)

Variable		N	%
**Sex**			
	Male	85	43.4
	Female	111	56.6
	Total	196	100
**Occupation**			
	Farmer	98	50
	Trader	41	20.9
	Teacher	15	7.6
	Student	33	16.8
	Unemployed	3	1.5
	Others	6	3.1
	Total	196	100
**Level of education**			
	Primary	44	22.4
	Secondary	86	43.9
	Tertiary	18	9.2
	None	48	24.5

Most of the participants without OAE were females, and farming was the most common income-generating activity.

### Prevalence of epilepsy

#### General prevalence

Fifty-three (53) persons (33 from Yangafock I and 20 from Yangafock II) met the ILAE criteria for epilepsy and the clinical criteria for onchocerciasis-associated epilepsy. For Yangafock I, the prevalence of epilepsy was 8.7% (78.75 per 1000), and for Yangafock II, it was 4.8% (47.7 per 1000).

#### Age specific prevalence

**Figure 3:**
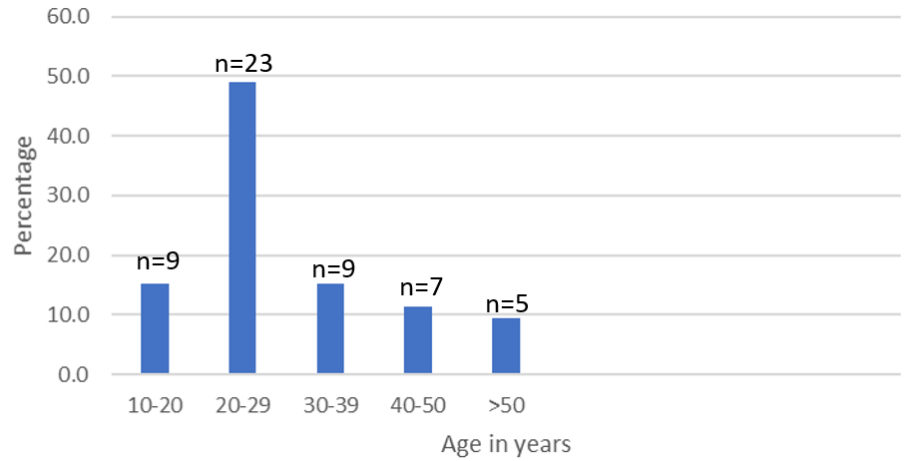
age specific prevalence of onchocerciasis-associated epilepsy cases

The most affected age group was 20-29 years, and the prevalence of epilepsy progressively decreased from the 30-39 age group until >50 years. Figure 3 displays the prevalence by age.

### Incidence

Nine cases in Yangafock I and 5 cases in Yangafock II had developed epilepsy within the five-year period (2017-2021) preceding the study, giving an incidence of epilepsy of 23.7 per 1000 and 11.9 per 1000 for Yangafock I and Yangafock II, respectively.

### Clinical characteristics of epilepsy

Table 4 shows the clinical characteristics of epilepsy in the study population. Most participants had their first seizure between the ages of 5 and 10 years. Generalized tonic-clonic epilepsy was the most common seizure type, and most patients experienced seizures 2-3 times per month.

**Table 4:** clinical characteristics of epilepsy among participants with onchocerciasis-associated epilepsy recruited from Yangafock I and II villages from May 1^st^ to May 31^st^, 2021 (N=53)

Variable		(%)	n
**Age of onset of epilepsy**			
	< 5 years	9.4	5
	5-10 years	52.8	28
	11-16 years	22.6	12
	> 16 years	15.1	8
	Total	100	53
**Duration of epilepsy**			
	< 5 years	7.5	4
	5-10 years	24.5	13
	> 10 years	67.9	36
	Total	100	53
**Seizure type**			
	Generalized tonic-clonic	86.8	46
	Absence	5.7	3
	Focal tonic-clonic with impaired awareness	3.8	2
	Focal secondarily generalized	3.8	2
	Total	100	53
**Seizure frequency**			
	0-1x per month	24.5	13
	2-3 per month	34	18
	Once per week	20.8	11
	2-4x per week	13.2	7
	5-6x per week	3.8	2
	Daily	3.8	2
	Total	100	53
**History of status epilepticus**			
	Yes	18.9	10
	No	81.1	43
	Total	100	53

### Community awareness of onchocerciasis-associated epilepsy

A total of 249 participants (including the 53 participants with OAE) completed the questionnaire for the assessment of community awareness. Table 5 summarizes the community responses to questions on OAE. Most of the participants had never heard that onchocerciasis can cause epilepsy. The majority believed that the high prevalence of epilepsy in their villages was due to witchcraft. Blindness was the most commonly cited complication of onchocerciasis. Most of the participants with OAE did not know if onchocerciasis was responsible for the epilepsy they had. A substantial proportion of participants without OAE believed they were not at risk of developing epilepsy if they contracted onchocerciasis. More than 60% of the participants without OAE stated that they would make more efforts to prevent onchocerciasis if they were aware that it causes epilepsy. Many of the participants said their attitude and behaviour towards people with epilepsy would change if they knew that epilepsies were caused by onchocerciasis.

**Table 5:** community awareness of onchocerciasis-associated epilepsy amongst participants with and without onchocerciasis-associated epilepsy recruited from Yangafock I and II villages from May 1^st^ to May 31^st^, 2021 (N=249)

Question	Frequency	%
**Have you ever heard that onchocerciasis can cause epilepsy?**		
No	228	91.57
Yes	21	8.43
**Total**	**249**	**100**
**In your opinion, what could be responsible for the high number of cases of epilepsy in your community?**		
Witchcraft	135	54.22
Ancestral curses	60	24.1
Onchocerciasis	8	3.21
I don’t know	45	18.47
**Which of the following is(are) complications of onchocerciasis?**		
Blindness	117	46.99
Skin disease	46	18.47
Epilepsy	0	0
Blindness and skin disease	80	32.13
All of the above	6	2.41
**(Persons with epilepsy only) In your opinion, do you believe that your illness has any link with onchocerciasis?**		
I don't know	32	60.4
No	18	33.96
Maybe	3	5.66
**(Persons with epilepsy only) In your opinion, do you believe that your seizures will reduce if you take Metizan?**		
I don't know	22	41.51
No	29	54.72
Maybe	2	3.77
**(Persons without epilepsy). In your opinion, do you believe that your risk of developing epilepsy would increase if you got infected with onchocerciasis?**		
No	129	65.82
Maybe	62	31.63
Yes	5	2.55
**Would you make more efforts to prevent onchocerciasis if you knew it could cause epilepsy?**		
Yes	161	64.66
May be	74	29.72
No	14	5.62
**Would your attitude towards people with epilepsy in your community change if you discovered that their epilepsy was caused by onchocerciasis?**		
Yes	74	37.75
Maybe	83	42.34
No	39	19.89
**Would you stop running away from people with epilepsy in your community if you knew that their epilepsy was caused by onchocerciasis?**		
Yes	70	35.71
May be	87	44.39
No	39	19.89
**Would you be more accepting of people with epilepsy in your community if you knew that their epilepsies was caused by onchocerciasis?**		
Yes	72	36.73
May be	85	43.37
No	39	19.89

## Discussion

We found a concerning low level of community awareness of OAE. While a lot has been done to increase awareness of the relationship between onchocerciasis and epilepsy among health professionals and scientists, these efforts may not have been translated into adequate community-level understanding. Our findings suggest that OAE knowledge has not yet permeated the communities most affected by it. This may act as a significant barrier to addressing the burden of this neglected tropical disease and its neurological sequelae. The implications of this finding are manifold. Firstly, a low level of awareness implies that many patients living with OAE may fail to seek appropriate medical care or support [20]. This can lead to no or suboptimal management of seizures and other symptoms and negatively impact their quality of life [20,21]. Moreover, a limited community understanding of the relationship between onchocerciasis and epilepsy can impede public health efforts to prevent and control onchocerciasis through vector management and mass drug administration [21].

Improving awareness of OAE at community level is thus crucial to the mitigation of the burden of not only OAE but also onchocerciasis [2,7,21]. Targeted educational campaigns, integrated within existing onchocerciasis and epilepsy control programs, could help bridge this knowledge gap at the community level [8,21]. Community outreach efforts should emphasize the causal link between onchocerciasis and epilepsy, as well as the availability of effective treatments and management strategies. Alongside these efforts, it is equally important to challenge the stigma and discrimination often faced by people with epilepsy in affected communities [7,21]. Integrating community education on OAE into existing public health initiatives could be a cost-effective and sustainable strategy [7,21].

We also found that a significant number of participants without OAE would change their attitudes and behaviours toward people with epilepsy if they knew the condition was linked to onchocerciasis. This suggests that increasing awareness could have a tangible impact on reducing stigma and improving social integration for those affected. Additionally, this highlights the need for a holistic approach that addresses not only the medical management of OAE but also the psychosocial and community-level factors that contribute to poor outcomes.

Ivermectin has been shown to effectively reduce the incidence of OAE as well as the frequency and severity of seizures in OAE in many onchocerciasis-endemic areas. A decrease in epilepsy prevalence with age-shift after ivermectin administration has been reported in previous studies [14,15]. In our study, participants with OAE did not believe that their seizure control could be improved by ivermectin. Knowledge of ivermectin's potential to prevent OAE could motivate increased compliance with mass drug administration campaigns.

The high prevalence of epilepsy in our study is consistent with findings from other rural communities in Cameroon [4,12,13] and other African countries where onchocerciasis is endemic [5-9]. The study sites' close proximity to fast-flowing rivers such as the Mbam, Ngoro, Mbi, and Kenkeng rivers puts the local population at a high risk of contracting onchocerciasis from black flies, the vector of O. vulvolus. Constant exposure of the local inhabitants to the high black fly biting rate can lead to increased viral load in the blood and an increased risk of brain injury, seizures, and epilepsy. A cohort study conducted by Chesnais *et al*. (2018) in the Mbam Valley showed that the risk of developing OAE later in life increased with a higher community viral load. Other studies conducted in the Mbam valley and other African countries, including systematic reviews with metanalysis [10,11] have confirmed the relationship between microfilaria load and increased risk of epilepsy. Another study in the Mbam and Sanaga river valleys of Cameroon reported a crude prevalence of epilepsy of 4.6% in one village and 7.8% in another, which is similar to the prevalence of OAE in our study [4].

The close proximity of Yangafock I to the Mbam River (Figure 1), which implies that the inhabitants of Yangafock I are closest to the black fly's breeding ground, could be the reason for the high prevalence and incidence of OAE in Yangafock I compared to Yangafock II. As a result, the village's inhabitants face a higher risk of contracting *O. volvulus*, leading to higher community microfilaria loads (CMFL), and consequently, a higher risk of developing epilepsy. Boussinesq *et al*. (2002) reported similar findings in a case-control study in the Mbam Valley. In the study, the researchers reported that the prevalence of epilepsy increased with increasing community microfilaria load and that the closer the village was to the Mbam River, the higher the prevalence of epilepsy [22].

The highest prevalence among youths between the ages of 20-29 years in our study is consistent with that reported by similar studies in Cameroon [4,22] and other African countries [5-9,11]. Youths aged between 20-29 years constitute the most active group in the study sites. They were actively involved in fishing, swimming, and sand extraction from the rivers compared to other age groups. These activities increase their exposure to black flies, which in turn increases their risk of fly bites, onchocerciasis, and OAE compared to other age groups. Furthermore, an inconsistent supply of medications often limits optimal treatment for epilepsy in rural communities. Patients might sometimes have the money to purchase the medications, but they are not available [21]. This lack of access to diagnostic services and specialized care hinders the ability to accurately diagnose and effectively treat epilepsy, resulting in a higher prevalence of the disease.

Evidence from our study underscores the need to improve community awareness of the relationship between onchocerciasis and epilepsy. Furthermore, our results strengthen the currently available evidence suggesting a causal relationship between onchocerciasis and epilepsy in onchocerciasis-endemic areas. Further studies to assess the ivermectin coverage and risk of transmission of onchocerciasis as well as the impact of ivermectin on the prevention and management of OAE in the study sites are warranted. The results from our study highlight the need to reinforce current strategies for onchocerciasis elimination and epilepsy management in rural communities.

### Limitations

In our study, we did not determine the relationship between CMFL density and risk/severity of epilepsy. Additionally, no brain imaging tests were done to identify other causes of epilepsy. We, however thoroughly screened for the presence of other risk factors by asking the necessary questions from the participants, their parents, caregivers, or guardians during the interrogative phase. We also reviewed past medical records (where available) and performed thorough clinical assessments for each participant to rule out epilepsy due to other courses. We conducted this study in only two communities, which limits the generalizability of the findings.

## Conclusion

Despite being a significant public health problem in many communities, community awareness of OAE is very low. Raising community awareness is critical to reducing the impact of OAE in high-prevalence communities.

### What is known about this topic

- Epilepsy is a public health problem in onchocerciasis-endemic areas;
- Onchocerciasis causes epilepsy.

### What this study adds

- Data on the neuroepidemiology of onchocerciasis-associated epilepsy in an onchocerciasis-endemic community that has not been previously investigated;
- The low level of community awareness of OAE in onchocerciasis-endemic communities with high prevalence of OAE underscores the need for awareness-raising and education at community level, efforts to increase awareness of the causal relationship between onchocerciasis and epilepsy should not only be concentrated on the scientific or healthcare community but should also be directed towards communities where onchocerciasis is endemic;
- Our study also shows that community engagement in the fight against onchocerciasis as well as community attitudes towards people with epilepsy in onchocerciasis-endemic areas can be improved if communities are educated about the causal relationship between onchocerciasis and epilepsy.

## References

[1] Burnham G (1998). Onchocerciasis. Lancet.

[2] Colebunders R, Njamnshi AK, van Oijen M, Mukendi D, Kashama JM, Mandro M (2017). Onchocerciasis-associated epilepsy: From recent epidemiological and clinical findings to policy implications. Epilepsia Open.

[3] Colebunders R, Siewe Fodjo JN, Hopkins A, Hotterbeekx A, Lakwo TL, Kalinga A (2019). From river blindness to river epilepsy: Implications for onchocerciasis elimination programmes. PLoS Negl Trop Dis.

[4] Siewe Fodjo JN, Tatah G, Tabah EN, Ngarka L, Nfor LN, Chokote SE (2018). Epidemiology of onchocerciasis-associated epilepsy in the Mbam and Sanaga river valleys of Cameroon: impact of more than 13 years of ivermectin. Infect Dis Poverty.

[5] Colebunders R, Y Carter J, Olore PC, Puok K, Bhattacharyya S, Menon S (2018). High prevalence of onchocerciasis-associated epilepsy in villages in Maridi County, Republic of South Sudan: A community-based survey. Seizure.

[6] Colebunders R, Mandro M, Njamnshi AK, Boussinesq M, Hotterbeekx A, Kamgno J (2018). Report of the first international workshop on onchocerciasis-associated epilepsy. Infect Dis Poverty.

[7] Hadermann A, Amaral LJ, Van Cutsem G, Siewe Fodjo JN, Colebunders R (2023). Onchocerciasis-associated epilepsy: an update and future perspectives. Trends Parasitol.

[8] Amaral LJ, Bhwana D, Fomo MF, Mmbando BP, Chigoho CN, Colebunders R (2023). Quality of life of persons with epilepsy in Mahenge, an onchocerciasis-endemic area in Tanzania: A cross-sectional study. Epilepsy Behav.

[9] Colebunders R, Hotterbeekx A, Siewe J, Mandro M, Mbonye M, Suykerbuyk P (2018). Epilepsy caused by onchocerciasis is an important public health problem in Africa. International Journal of Infectious Diseases.

[10] Kaiser C, Pion SD, Boussinesq M (2013). Case-control studies on the relationship between onchocerciasis and epilepsy: systematic review and meta-analysis. PLoS Negl Trop Dis.

[11] Pion SD, Kaiser C, Boutros-Toni F, Cournil A, Taylor MM, Meredith SE (2009). Epilepsy in onchocerciasis endemic areas: systematic review and meta-analysis of population-based surveys. PLoS Negl Trop Dis.

[12] Chesnais CB, Nana-Djeunga HC, Njamnshi AK, Lenou-Nanga CG, Boullé C, Bissek AC (2018). The temporal relationship between onchocerciasis and epilepsy: a population-based cohort study. The Lancet Infectious Diseases.

[13] Chesnais CB, Bizet C, Campillo JT, Njamnshi WY, Bopda J, Nwane P (2020). A Second Population-Based Cohort Study in Cameroon Confirms the Temporal Relationship Between Onchocerciasis and Epilepsy. Open Forum Infect Dis.

[14] Boullé C, Njamnshi AK, Dema F, Mengnjo MK, Siewe Fodjo JN, Bissek AZ (2019). Impact of 19 years of mass drug administration with ivermectin on epilepsy burden in a hyperendemic onchocerciasis area in Cameroon. Parasit Vectors.

[15] Dusabimana A, Tsebeni Wafula S, Raimon SJ, Fodjo JNS, Bhwana D, Tepage F (2020). Effect of Ivermectin Treatment on the Frequency of Seizures in Persons with Epilepsy Infected with Onchocerca volvulus. Pathogens.

[16] Weldegebreal F, Medhin G, Weldegebriel Z, Legesse M (2014). Assessment of community's knowledge, attitude and practice about onchocerciasis and community directed treatment with Ivermectin in Quara District, north western Ethiopia. Parasit Vectors.

[17] Domche A, Nana-Djeunga HC, Yemeli LD, Nanga CL, Boussinesq M, Njiokou F (2021). Knowledge/perception and attitude/practices of populations of two first-line communities of the Centre Region of Cameroon regarding onchocerciasis and black fly nuisance and bio-ecology. Parasit Vectors.

[18] Diagana M, Preux PM, Tuillas M, Ould Hamady A, Druet-Cabanac M (2006). Dépistage de l'épilepsie en zones tropicales: validation d'un questionnaire en Mauritanie [Dépistage de l'épilepsie en zones tropicales: validation d'un questionnaire en Mauritanie]. Bull Soc Pathol Exot.

[19] Fisher RS, Cross JH, French JA, Higurashi N, Hirsch E, Jansen FE (2017). Operational classification of seizure types by the International League Against Epilepsy: Position Paper of the ILAE Commission for Classification and Terminology. Epilepsia.

[20] Jada SR, Tionga MS, Siewe Fodjo JN, Carter JY, Logora MY, Colebunders R (2022). Community perception of epilepsy and its treatment in onchocerciasis-endemic villages of Maridi county, western equatoria state, South Sudan. Epilepsy Behav.

[21] Siewe Fodjo JN, Dekker MCJ, Idro R, Mandro MN, Preux PM, Njamnshi AK (2019). Comprehensive management of epilepsy in onchocerciasis-endemic areas: lessons learnt from community-based surveys. Infect Dis Poverty.

[22] Boussinesq M, Pion SD, Demanga-Ngangue Kamgno J (2002). Relationship between onchocerciasis and epilepsy: a matched case-control study in the Mbam Valley, Republic of Cameroon. Trans R Soc Trop Med Hyg.

